# Menarcheal Age and Risk of Type 2 Diabetes: A Community-Based Cohort Study

**DOI:** 10.4274/jcrpe.3370

**Published:** 2017-06-01

**Authors:** Maryam Farahmand, Fahimeh Ramezani Tehrani, Marzieh Rostami Dovom, Fereidoun Azizi

**Affiliations:** 1 Reproductive Endocrinology Research Center, Research Institute for Endocrine Sciences, Shahid Beheshti University of Medical Sciences, Tehran, Iran; 2 Endocrine Research Center, Research Institute for Endocrine Sciences, Shahid Beheshti University of Medical Sciences, Tehran, Iran

**Keywords:** Menarcheal age, blood glucose, reproductive age, noncommunicable disease

## Abstract

**Objective:**

It has been reported that early menarche is associated with higher risk for type 2 diabetes. We aimed to explore the association between age at menarche and risk of type 2 diabetes in a population-based cohort study.

**Methods:**

For the purpose of the present study, 5191 subjects of reproductive age who were participants of the Tehran Lipid and Glucose Study and also met the eligibility criteria were selected. Demographic, lifestyle, reproductive, and anthropometric data as well as risk factors for metabolic diseases were collected. Menarcheal age was categorized into five categories, as <11 years, 11-12 years, 13-14 years, 15-16 years, and >17 years. Diabetes and pre-diabetes were defined according to the American Diabetes Association criteria. Logistic regression analysis was used to assess the risk of the menarcheal age group for type 2 diabetes and pre-diabetes.

**Results:**

Of 5625 participants, 673 women had pre-diabetes and 187 had diabetes. Early menarche was associated with higher risk of diabetes and pre-diabetes, compared to the reference group (13-14 years), (OR=3.55, 95% CI: 1.6-7.8 and OR=2.55, 95% CI:1.4-4.8, respectively), an association which remained after further adjustment for potential confounders including family history of diabetes, parity, education, age, body mass index, waist circumference, smoking history, physical activity, and duration of oral contraceptives use.

**Conclusion:**

Results showed early menarche to be a potential risk factor for type 2 diabetes and pre-diabetes.

## What is already known on this topic?

Results indicate that early menarche is a potential risk factor for type 2 diabetes and pre-diabetes.

## What this study adds?

Results on a large cohort-supported previous findings indicating that early menarche is a potential risk factor for type 2 diabetes and pre-diabetes.

## INTRODUCTION

Puberty is considered to be a reproductive milestone in a woman’s life (1,2). The initiation of puberty is strongly regulated by genes, and early puberty may be associated with an increased risk for various poor health outcomes, including obesity, type 2 diabetes, cardio-vascular disease (3,4,5).

Assessment of menarcheal age, namely, age at the initiation of menstruation, enables researchers, using a noninvasive approach, to investigate the association between developmental timing and future disease states (6). Recent studies demonstrate that obesity, type 2 diabetes, and even cardiovascular mortality may all be related to early pubertal maturation (7).

Results of studies show a relationship between prevalence of type 2 diabetes and pubertal timing, an association, which is attenuated when adjusting for adulthood body mass index (BMI) (4,5,8).

The prevalence of type 2 diabetes has increased rapidly worldwide during the recent decades (4,5). At the time of their diagnosis for type 2 diabetes, a number of individuals have been shown to have already developed important complications, a finding making it increasingly important to identify those at risk in early life, especially when there is a possibility of early intervention (4,9).

Individuals with a pre-diabetes status, namely, those with an impaired fasting glucose and/or impaired glucose tolerance, are at the highest risk of developing type 2 diabetes (10). Unfortunately, data on the effects of lifestyle or therapeutic interventions for the primary prevention of pre-diabetes are still in an inadequate stage (11).

On the other hand, studies have suggested that earlier menarche is related to poorer glycemic control in later life, to increased risk factors for diabetes such as excessive adiposity in childhood (12,13,14) and adulthood (15,16,17), as well as to elevated blood glucose levels (18) and insulin resistance (19), independent of adiposity (4).

Concurrent with the rising prevalence of obesity, menarcheal age in Iranian girls has been declining during recent decades (20,21,22).

This study was conducted to investigate the association between early menarcheal age and risk of type 2 diabetes and pre-diabetes in a population-based cohort study of the Tehran Lipid and Glucose Study (TLGS).

## METHODS

TLGS is an ongoing prospective study initiated in 1998 with the aim of determining the prevalence of non-communicable disease risk factors. The project is conducted on a representative sample of residents of District 13 of Tehran, the capital of Iran. Details of the rationale of the study have been published elsewhere (23). The participants in the study consisted of 15 005 individuals (male and female) aged ≥3 years. Of the 7718 females (aged 10-50 years), 5625 met our eligibility criteria (having complete data and not having yet reached menopause), after exclusion of those with pathological late-onset puberty such as in case of hypothyroidism (n=3), Cushing’s syndrome (n=0), hypopituitarism (n=0), chronic renal failure (n=1), type one diabetes (n=2).

The ethical committee of the Research Institute for Endocrine Sciences approved this study. Written informed consent was obtained from all study participants.

For all participants, demographic and lifestyle variables as well as information on various risk factors for non-communicable diseases and their medical and reproductive histories were collected by trained interviewers, during face-to-face interviews.

A modifiable activity questionnaire was used to assess the achieved physical activity pattern (24); the subjects were asked to report the physical activities in which they had participated during the past 12 months. “Leisure time physical activity” was defined as performing three or more days of vigorous-intensity activity of at least 20 minutes, or five or more days of moderate-intensity activity or walking at least 30 minutes, or five or more days of any combination of walking, moderate or vigorous-intensity activities, achieving a minimum of at least 600 metabolic equivalent task minutes per week (25,26).

Height was measured with a measuring tape with a 0.5-cm accuracy, in a standing position against a wall, without shoes, and with shoulders in a normal position. Weight was measured by an electronic digital weighing scale with 100-g accuracy while the subject was minimally clothed and without shoes. BMI was calculated by dividing weight in kilograms by height in meters squared. Waist circumference (WC) was measured midway between the lower rib margin and the iliac crest at the end of a gentle expiration with 0.5-cm accuracy and without any compulsory pressure. Blood samples were taken after a 12-h overnight fast for biochemical measurements.

### Definitions

Menarcheal age was defined as the age at the first menstrual bleeding, based on data obtained during interviews with participants. For our analysis, menarcheal age was categorized into five categories including <11 years, 11-12 years, 13-14 years, 15-16 years, and >17 years.

Diabetes was defined according to the American Diabetes Association (2013), namely as a fasting blood glucose ≥126 mg/dL (7.0 mmol/L) or a 2 h plasma glucose ≥200 mg/dL (11.1 mmol/L) (27).

Pre-diabetes was defined according to the American Diabetes Association (2013) as a fasting plasma glucose of 100 mg/dL (5.6 mmol/L) to 125 mg/dL (6.9 mmol/L) or a impaired fasting glucose or 2-h PG of 140 mg/dL (7.8 mmol/L) to 199 mg/dL (11.0 mmol/L) (impaired glucose tolerance) (27) in the 75-g oral glucose tolerance test or drug treatment.

### Laboratory Tests

All blood analyses were done at the TLGS research laboratory on the day of blood collection. Plasma glucose was measured by the enzymatic colorimetric method, using a glucose oxidase kit (Pars Azmoon Inc., Tehran, Iran); inter- and intra-assay coefficients of variation were both <2.2%.

### Details of Ethics Approval

The ethical review board of the Research Institute for Endocrine Sciences approved the study proposal, and written informed consent was obtained from all subjects.

### Data Analysis

Demographic and reproductive characteristics were compared by menarcheal age in participants, using ANOVA test for continuous variables and Dunnett post-hoc test and χ^2^ test for categorical variables.

The logistic regression method was used to assess the risk of the menarcheal age group (independent variable) for type 2 diabetes and pre-diabetes (dependent variables), before and after adjustment for confounding variables including family history of diabetes, parity, education, age, BMI, WC, smoking history, physical activity, and duration of oral contraceptives (OCP) use. The reference was women with a menarcheal age of >13 and ≤14 years, as this group constituted 49.4% of all participants.

Data were analyzed using SPSS 15 (SPSS Inc., Chicago, IL, USA).

## RESULTS

Among 5625 participants, 673 women had pre-diabetes and 187 had diabetes. The demographic and reproductive characteristics of the participants stratified by five menarcheal age groups are presented in Table 1. The number of women in each menarcheal age group were 109, 1603, 2779, 1027, and 107, respectively.

Results showed that the mean menarcheal age was 13.3±1.5 years; mean age (p=0.001), BMI (p=0.046), and WC (p=0.001) differed significantly between these groups. 

Logistic regression analysis demonstrated that there was a statistically significant difference in the risk of pre-diabetes type 2 between women with earlier menarche [<11 years (group 1)] and women with menarcheal ages of >13 and ≤14 years [reference (group 3)], before and after adjustment for covariates (Table 2); a statistically significant difference in the risk of diabetes type 2 between women with earlier menarche [<11 years (group 1)] and women with menarcheal ages of >13 years and <14 years [reference (group 3)] was also demonstrated before and after adjustment for covariates (Table 3). The reference group was women with menarcheal ages of >13 years and ≤14 years as it constituted 49.4% of all participants.

## DISCUSSION

In this population-based study, we found that early menarche (<11 years) was significantly associated with increased risk of type 2 diabetes and pre-diabetes before and after adjustment for potential confounders (Tables 2, 3). The potential confounders including family history of diabetes, parity, education, age, BMI, WC, smoking history, physical activity, and duration of OCPs use were adjusted in regression models.

The decreasing age at menarche among Iranian women (22) noted in recent decades is an issue of concern for future risk of type 2 diabetes and pre-diabetes. On the other hand, the main pathways explaining the association between early menarche with type 2 diabetes have not been well described. It has been shown that lower serum concentration of insulin-like growth factor-1 (IGF-1) is associated with an increase in type 2 diabetes (28).

Estrogen modulates growth hormone secretory activity in a biphasic manner; low levels of estrogen stimulate secretion of IGF-1 through growth hormone release, whereas high levels inhibit IGF-1 production resulting in menarche (29,30), indicating that early exposure to higher levels of estrogens may lead to diabetes in the future (31). Also the growth spurt and the beginning of menstruation during puberty are caused by estrogen secretion. The results of a study conducted among 329 girls showed that earlier menarche was related to lower levels of both IGF-binding protein-I and sex hormone-binding globulin (SHBG) and also to higher levels of IGF-I, androstenedione, dehydroepiandrosterone sulfate (DHEAS), leptin, and fasting insulin at the age of 8 years (32). Also, the associations between high levels of IGF-I, androstenedione, and DHEAS to earlier menarche continue after adjustment for BMI (32). Insulin resistance manifesting early in life could be an important pathologic disorder caused by association between earlier age at menarche and higher risk of diabetes (33).

There are limited studies on the association between menarcheal age and risk of type 2 diabetes and pre-diabetes and results of those available are controversial (2,34,35).

Results of the Stöckl et al (2) study, conducted on 1503 women aged 18-32 years, showed an adverse association between menarcheal age and diabetes/pre-diabetes, before and after adjusting for potential confounders. Gambineri et al (34), in their study conducted on 121 women with polycystic ovary syndrome, reported that early menarcheal age was associated with type 2 diabetes. On the other hand, the Rancho Bernardo Study of 997 post-menopausal women aged 50-92 years, showed that menarcheal age was not associated with abnormal glucose tolerance or type 2 diabetes, whereas late menarcheal age was inversely associated with fasting and post challenge glycemic levels (35). The Shanghai Women Health Study, conducted on 69385 women aged 40-70 years, reported that older age at menarche was significantly associated with reduced risk of diabetes after adjustment for birth cohort, education, and household income, a significance that disappeared after adjustment for baseline BMI (36). Another study, conducted on 34022 Chinese women aged 45-74 years, reported that older menarcheal age was related to lower prevalence of diabetes after adjusting for several confounders. Baseline BMI and menarcheal age of <12 years (compared to 13-14 years) was related to an 18% higher risk of diabetes, even after adjustment for BMI (33). Results of another study from China on postmenopausal women showed that early menarche was not associated with diabetes. Differences in the results obtained in these studies have been attributed to differences in menarcheal age grouping and participant recruitment (37). The results of the cohort study of the European Prospective Investigation into Cancer and Nutrition (the EPIC-Norfolk study) conducted on 13308 women aged 40-75years showed that menarcheal age was inversely related to diabetes, an effect totally induced by adult adiposity (38).

Also, the Atherosclerosis Risk Communities Study (ARIC), conducted among 8491 women aged 45-65 years (39) reported that age at menarche was inversely related to diabetes after adjusting for potential confounders; although these associations were partially reduced by adult adiposity, the association remained significant. On the other hand, in the Nurses’ Health Study conducted among 100547 younger women (26-46 years), those with earlier menarcheal age had raised risk of diabetes even after adjusting for adiposity, findings consistent with ours (4).

Several studies report that early menarche is associated with an increased risk of diabetes even after adjusting for potential confounders (33,37,38,39). Also, results of other studies showed an association between early menarcheal age and elevated fasting insulin, insulin resistance (HOMAIR), and A-cell function (HOMA-A), compared with usual menarcheal age (31,40,41). However, our results and those of some other studies yield additional evidence that early menarcheal age is related to risk of diabetes, although the mechanism(s) of this association are unclear (31).

Based on results of studies in US, Europe, China, and Iran, the secular trend of menarcheal age has decreased during the recent decades (22,42,43). Moreover, the prevalence of diabetes mellitus has increased worldwide sharply during these years (4,5) indicating that earlier menarcheal age is related to a diabetes risk although the fundamental mechanism of this association is still unknown (31).

In this population-based study, we found that early menarche (≤11 years) was significantly associated with increased risk of type 2 diabetes and pre-diabetes, a result which persisted after adjustment for potential confounders including family history of diabetes, parity, education, age, BMI, WC, physical activity, smoking history, and duration of OCPs use; risk of pre-diabetes increases even after further adjustment for BMI. This finding may be partly explained by the dual role of obesity in both early menarche and diabetes type 2. It has been shown that obesity is associated with early onset of puberty (40,44,45) as well as with an elevated risk of insulin resistance and type 2 diabetes (46). Fredriks et al (43) demonstrated that women with a history of early menarche have an increased risk of type 2 diabetes and that this is mainly due to the direct effect of early onset of puberty on the risk of diabetes; BMI has a limited effect on this association.

In this study, the duration of OCPs use was adjusted as a potential confounder because some studies have reported changes in carbohydrate metabolism in OCP users that are related to both estrogen and progesterone which are components of OCPs (47). Existing studies on the effects of OCPs on type 2 diabetes have demonstrated controversial results. There are studies reporting an increase in insulin resistance and fasting blood glucose (FBG) among OCPs users (47,48) In contrast, the results of another study showed that women with prolonged OCP use had FBG levels lower than never users (49). There are also studies reporting that the risk of type 2 diabetes did not differ between long-term OCP users and never users (50,51).

Regarding strengths and limitations, this is the first study demonstrating a significant association between earlier menarcheal age and raised risk of diabetes and pre-diabetes in a large Middle East population (n=5625); most studies reporting a relationship on such issues have been conducted in Western countries (31). Our study has the advantage of using an ongoing population-based cohort. The amount of intra-assay variability in our data is also likely to be minimal because all laboratory measurements were done simultaneously at the same laboratory, by the same person. However, the limitations of our study were that pre-pubertal information on our participants as well as measurements of HA1C were not available. We therefore added any treatment for diabetes as a criterion of presence of diabetes. Recall bias might be a problem with self-reporting of menarcheal age; however, in the TLGS cohort, menarcheal age was assessed four times (once every three years) and the findings showed good confirmation.

In conclusion, it can be stated that early menarche is associated with an increase in prevalence of type 2 diabetes. This risk factor needs to be considered in screening programs of diabetes conducted at a community level. Identification of individuals at higher risk and implementing adequate prevention programs may decrease the adverse consequences of diabetes resulting from micro- and macro-vascular complications (52,53).

## Figures and Tables

**Table 1:**
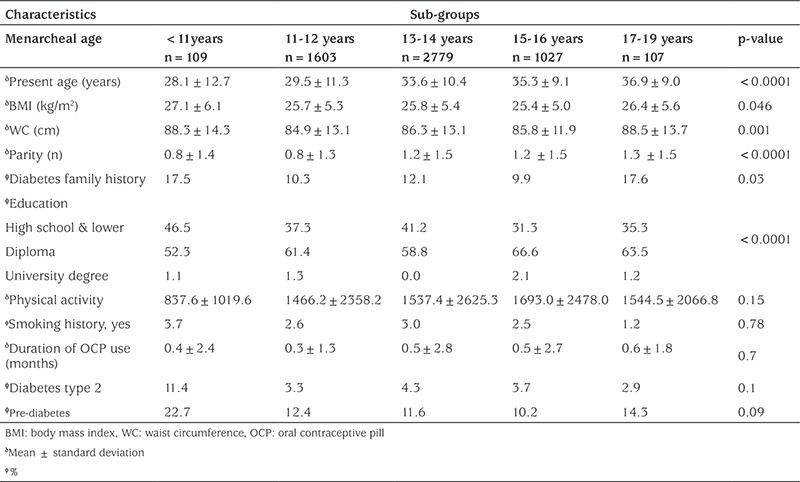
Characteristics of the study participants according to their menarcheal age

**Table 2:**
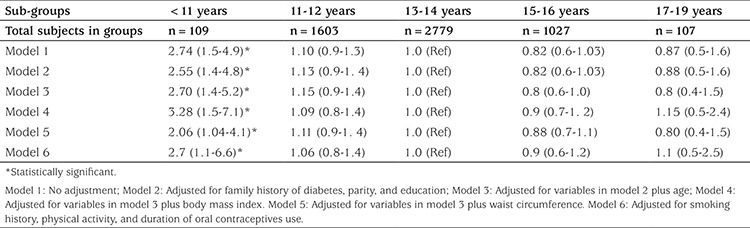
Risk ratios (and 95% confidence intervals) for pre-diabetes by menarcheal age

**Table 3:**
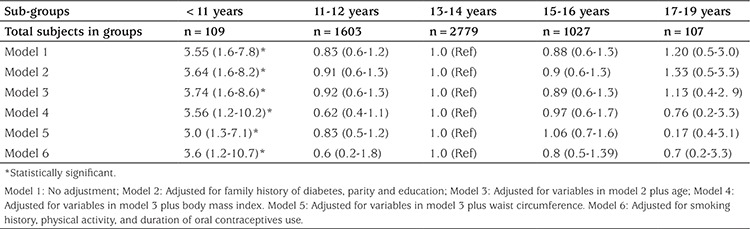
Risk ratios (and 95% confidence intervals) for type 2 diabetes by menarcheal age groups
